# Neovascular glaucoma: prevention and treatment

**Published:** 2022-01-31

**Authors:** Jibran Mohamed-Noriega, Jason A Penniecook

**Affiliations:** 1 Associate Professor: Department of Ophthalmology, University Hospital and Faculty of Medicine, Autonomous University of Nuevo Leon (UANL), Monterrey, Mexico.; 2 Ophthalmologist (Glaucoma): Instituto de la Visión, Centro Mexicano de Salud Visual Preventiva, Collaborative Network for Quality in Eye Research, Montemorelos, Mexico.

**Patients with diabetic retinopathy and retinal vein occlusion are at risk of developing neovascular glaucoma, a blinding and painful condition. Early detection and prompt treatment is vital.**

Neovascular glaucoma (NVG) is a devastating type of glaucoma caused when new and abnormal blood vessels block the trabecular meshwork (the tissue that drains fluid out of the eye). The formation of new blood vessels is most frequently caused by diabetic retinopathy (DR) and retinal vein occlusion. However, other atypical causes also need to be considered (see panel).

## Presentation

In the early stages of this condition, patients present with symptoms of the underlying disease (diabetic retinopathy is the most common cause) such as blurred vision, floaters, or complete vision loss due to a bleed in the back of the eye (retinal or vitreous haemorrhage). As the disease progresses and the intraocular pressure (IOP)increases, patients can develop severe pain in the eye, headache, a red eye, nausea, or vomiting.

**Atypical causes of neovascular glaucoma**

1. In children, retinoblastoma and Coates disease should be excluded.
2. If there is no retinal vein occlusion or bilateral DR, consider ocular ischaemic syndrome and ask for a Doppler ultrasound of the carotid arteries.
3. If all of the above are excluded, consider uveitis, other intraocular tumours, or peripheral retinal diseases.

**Figure 1:**
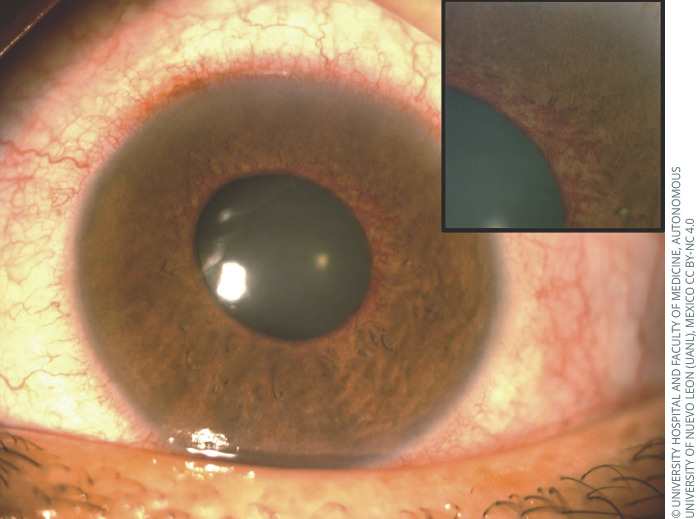
A diabetic patient that presented with severe pain, blurred vision and red eye. Signs of the acute stage of neovascular glaucoma are seen in this photo: ciliary injection, mild corneal epithelial oedema, and rubeosis iridis. *Top right:* An enlarged view of rubeosis iridis.

On examination, blood vessels can be seen around the pupil (rubeosis iridis); see Figure 1. Large vessels can be detected using a torch, but smaller vessels can be difficult to detect in the early stages, even when using a slit lamp. In order to detect rubeosis iridis early, it is therefore important to **examine the iris before dilation**.

The anterior chamber angle is open in the early stages of neovascular glaucoma. However, as the new vessels grow, peripheral anterior synechiae (adhesions of iris to the cornea) develop and these can close the angle, resulting in further increases in IOP. The pupil also tends to be less reactive to light and eventually progresses to a fixed, dilated pupil with abnormal curling of the more pigmented layer of the iris around the pupil margin (ectropion uveae). The new blood vessels are particularly fragile and sometimes bleed spontaneously in the anterior chamber (hyphema).

## Natural history

Disease processes in the eye (DR, retinal vein occlusion, or others) trigger the production of vasoproliferative factors such as vascular endothelial growth factor (VEGF), which in turn promotes the formation of new, fragile blood vessels that are prone to leaking or bleeding. The new blood vessels (Figure 1) appear at the pupil margin and/or anterior chamber angle. Initally, the IOP is normal as the new vessels only partially block the angle; not enough to obstruct outflow and increase the IOP.

As the vessels continue to grow, aqueous outflow is reduced and the IOP increases, although the angle remains open. Eventually, contractile cells are formed and they cause the iris to adhere to the inner part of the cornea in the periphery of the iris (peripheral anterior synechiae), eventually completely obstructing the trabecular meshwork and inducing ectropion uveae at the pupillary border. The anatomical blockage of the trabecular meshwork progresses to a complete closure of the angle (the angle is drawn together like a zipper), and the IOP increases to very high levels. As a result, the corneal endothelial cells cannot maintain the cornea’s transparency and corneal oedema appears, visible as a hazy cornea. The breakdown of the blood-aqueous barrier produces anterior chamber flare and inflammation is seen clinically as ciliary injection and anterior chamber cells.

The patient will experience pain, headache, nausea, and vomiting over days to weeks. The pronounced increase in intraocular pressure also damages the optic nerve, with progressive sight loss in the affected eye. In some cases, the ciliary body becomes progressively ischaemic to the point that it no longer produces aqueous humour and some eyes can then progress to phthisis bulbi (a shrunken, non-functioning eye).

In many patients with diabetes, the severity of the neovascular glaucoma may not mirror the severity of the diabetic disease seen on examination of the eye. For instance, many patients with proliferative DR may have normal IOP and no anterior segment neovascularisation. On the other hand, patients with normal visual acuity may have proliferative DR and severe neovascular glaucoma with pain as the first clinical sign that they have diabetic eye disease. Therefore, the clinicians who assess patients with diabetes or other retinal vascular diseases should be familiar with neovascular glaucoma and examine patients to assess for neovascularisation of the anterior segment. Patients with neovascular glaucoma should be referred urgently to ophthalmologists trained in treating glaucoma and the underlying retinal causes.

## Detection

When examining a patient with, or suspected to have, neovascular glaucoma, ask yourself the following questions.

1. Is the diagnosis neovascular glaucoma, or another type of secondary glaucoma?
2. What is the the underlying disease that caused the neovascular glaucoma? The cause is often DR or retinal vein occlusion, but also rule out atypical causes (see panel on p. 10).
3. How high is the IOP? It can be considered severe when the patient develops signs or symptoms of very high IOP (corneal oedema, ocular pain, or headaches).
4. Is the angle opened or closed? See article on gonioscopy within this issue of the journal.
5. What is the visual potential? It can be roughly estimated based on the severity of the macular damage caused by the underlying disease and the damage to the optic nerve caused by glaucoma.
6. What is the life expectancy of the patient?
7. Is the patient in pain or comfortable?
8. Are there underlying systemic diseases that require an urgent referral, such as renal failure or cancer?

Involve the other health care providers who are required to treat the patient (primary care doctor, nephrologist, cardiologist, neurologist, or nutritionist). Advise patients and carers to avoid the overuse of non-steroidal anti-inflammatories (NSAIDs) for pain relief due to the risk of gastrointestinal bleeding.

## Treatment

Treatment is challenging and requires close collaboration between different health professionals. Treatment will often involve a combination of medical treatment as well as laser or surgical treatment (see Table 1).

**Table 1:** Treatment of neovascular glaucoma according to clinical manifestations

Characteristics	IOP	Treatment to control IOP	Treatment to control neovascularization
New blood vessels (rubeosis iridis) on the iris or anterior chamber angle	Normal	No	Pan-retinal photocoagulation, retinal cryotherapy, or intravitreal anti-VEGF
Rubeosis iridis with an open angle	High	IOP-lowering drops, if no improvement surgery	
Rubeosis iridis with a closed angle	High	Surgery	
Severe neovascular glaucoma (with severe pain or IOP > 40 mmHg at presentation)	Very high	**Urgent surgery** to lower the IOP, such as a glaucoma drainage device or a cyclodestructive procedure. Intravitreal anti-VEGF, pan-retinal photocoagulation or retinal cryotherapy will also be needed.	
End-stage (blind) neovascular glaucoma (no perception of light)	Low to very high	Usually for pain control only, e.g., steroids and cycloplegic eye drops, as well as laser or surgery to lower the IOP.	

When the angle is still open, early treatment with pan-retinal photocoagulation and intravitreal anti-VEGF may cause regression of the neovascularisation and a return of IOP to normal. On the contrary, when the angle is closed 360 degrees (zipped), almost all patients will require surgery as their natural outflow cannot be improved. Clinicians should be aware that an intravitreal injection may itself increase the eye pressure. Abrupt reduction of IOP with a paracentesis is not advisable, due to the risk of hyphaema, vitreous haemorrhage, or decompression retinopathy.

**How to prevent neovascular glaucoma**

All health care providers (doctors, nurses, technicians, nutritionists, health visitors, etc.) should encourage patients to actively control any pre-exisiting conditions, such as diabetes or hypertension.

1. Encourage patients with diabetes to control their blood sugar levels. Elevated blood sugar levels encourage the formation of the abnormal blood vessels.
2. Advise all patients to have annual eye examinations (more frequent examinations may be required).
3. Promptly treat severe or proliferative DR with pan-retinal photocoagulation (PRP)
4. In patients with retinal vein occlusion, carry out slit lamp examination and gonioscopy to monitor the anterior segment closely (monthly if ischaemic). Look out for iris or anterior chamber angle neovascularisation during the first 3–6 months.
5. If regular anti-VEGF intravitreal injections are used to treat DR or retinal vein occlusion, monitor the patient’s eye closely for 3–6 months after treatment is stopped.
6. Monitor the severity of DR closely after cataract surgery, particularly if there was a posterior capsule rupture during surgery.

### Medical treatment

Medical treatment should include steroid, cycloplegic, and IOP-lowering eye drops. Acetazolamide is useful, but it should be prescribed carefully because many patients have renal failure and will be on multiple medications. It should also be avoided in patients with sickle cell disease.

### Surgical treatment

The type of surgical management for raised IOP will depend on the visual prognosis and life expectancy. For patients with poor visual potential, an aggressive cyclodestructive procedure with cryotherapy or diode laser can effectively reduce symptoms and partially stabilise the IOP (sometimes it is easier to combine this procedure with peripheral retinal ablation using the same cryotherapy or diode laser).

For patients with good visual potential, an alternative to a cyclodestructive procedure is a glaucoma drainage device, such as an Ahmed or Baerveldt device. The choice of intervention for IOP reduction should consider the presumed life expectancy of the patient and the effects of treatment on the patient’s quality of life, all costs involved (eye drops, surgery, transport, carer availability, time off work), and the patient’s beliefs and preferences.

**Blind eyes** should only be treated to control pain (see the article on treating the painful blind eye). It should be emphasised that neovascular glaucoma secondary to DR may be asymmetrical, but tends to be bilateral. **The treatment of neovascular glaucoma in the worst eye should not distract clinicians from treating the better eye with pan-retinal photocoagulation.**

